# Targeting macrophage endocytosis via platelet membrane coating for advanced osteoimmunomodulation

**DOI:** 10.1016/j.isci.2022.105196

**Published:** 2022-09-23

**Authors:** Wendong Gao, Lan Xiao, Yuqing Mu, Yin Xiao

**Affiliations:** 1 Centre for Biomedical Technologies, Queensland University of Technology (QUT), Brisbane, QLD 4059, Australia; 2 The Australia-China Centre for Tissue Engineering and Regenerative Medicine (ACCTERM), Queensland University of Technology (QUT), Brisbane, QLD 4000, Australia; 3 Key Laboratory of Oral Medicine, Guangzhou Institute of Oral Disease, Stomatology Hospital of Guangzhou Medical University, Guangzhou 510140, China; 4 School of Medicine and Dentistry, Griffith University, Gold Coast Campus, Brisbane, QLD 4222, Australia

**Keywords:** Biological sciences, Drug delivery system, Health sciences, Immunology, Medicine

## Abstract

The identification, uptake, and clearance of nanoparticles (NPs) by phagocytes are critical in NP-based therapeutics. The cell membrane coating technique has recently emerged as an ideal surface modification approach to help NP bypass phagocytosis. CD47, a regulatory protein for phagocytosis, is a cell surface glycoprotein expressed on all cell types, including platelets. Herein, we enclosed bioactive glass (BG) with a platelet membrane to bestow BG with unique cell surface functions for immune evasion and immunomodulation. Compared with the uncoated particles, platelet membrane-coated BG shows reduced cellular uptake and can generate an immune environment favorable for osteogenesis. This is evidenced by the triggering of robust osteogenic differentiation in bone mesenchymal stromal cells, suggesting the synergistic effect of platelet membrane and BG in bone regeneration. These collectively indicate that cell membrane coating is a promising approach to enhance the therapeutic efficacy of biomaterials and thus provide new insight into biomaterial-mediated bone regeneration.

## Highlights

• Platelet membrane with key protein has been successfully coated on bioactive glass.
• Platelet membrane-coated bioactive glass (PBG) can reduce the uptake by macrophages.
• PBG generates an immune environment favorable for osteogenesis.

## Graphical abstract

**Figure:**
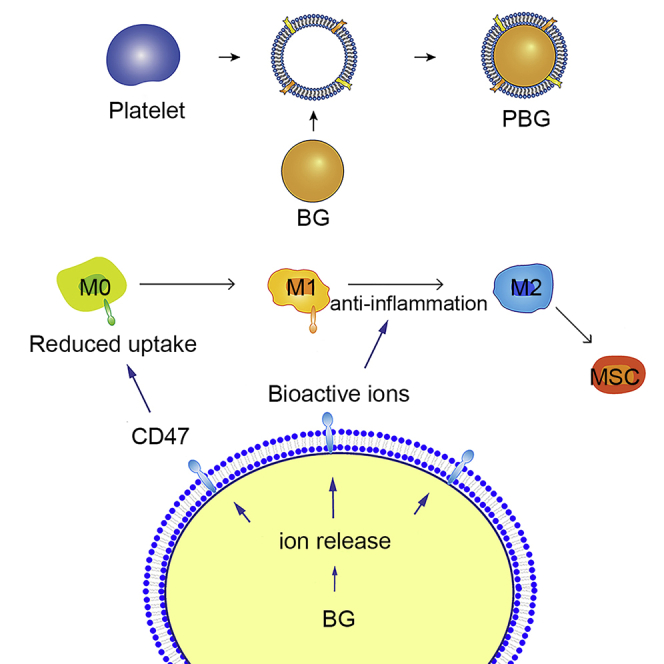

## Introduction

The design and application of nanoscale materials for disease treatment represent an important field of current biomedical research (Mitchell et al., 2021), in which nanomaterials are primally engineered as delivery vehicles or therapeutic agents to improve clinical practice outcomes (Gao and Xiao, 2022). The efficient delivery of nanomaterials to disease sites *in vivo* requires complete control over the nanomaterial transport in cells, tissues, and the body (Kumar et al., 2021; Sun et al., 2022). However, as foreign invaders, nanomaterials are typically recognized and uptaken by phagocytes to trigger significant immune responses, leading to reduced delivery efficacy and dysregulated immune microenvironment (Gustafson et al., 2015). Therefore, controlling the clearance of nanomaterials by phagocytes is yet one of the most significant challenges in the clinical translation of nanomaterials. So far, various bottom-up strategies, such as polymer grafting (Liu et al., 2014), biomolecule modification (Krishnamurthy et al., 2019), and lipid coating (Liu et al., 2009), have been developed to reduce nanomaterial clearance by the mononuclear phagocytic system (MPS). However, accumulating evidence suggests that current strategies are compromised. For example, polyethylene glycol (PEG) coating would unavoidably induce immune responses and protein adsorption during the *in vivo* circulation, despite it having been used as a standard approach for many years (Adamiak et al., 2017; Anselmo et al., 2015).

To strengthen the benefits of nanomaterials while alleviating their side effects in clinical application, biomaterials that mimic the structural and biological features of natural tissues have been intensively studied and developed in recent years, among which cell membrane-coating technology has emerged as a novel approach to impart materials with cell-like abilities (Stephan et al., 2010; Zinger et al., 2020). As one of the most basic life forms, cells can carry out many essential functions (Heath, 2000), a significant portion of which is directed by cell membranes through the embedded biomacromolecules (Bretscher, 1985; Fang et al., 2017). To date, various cell membranes have been used as bio-stealth materials to enhance the *in vivo* performance of nanomaterials, and such an approach achieved promising outcomes in biomedical applications such as drug delivery (Thanuja et al., 2018), anti-infection (Rao et al., 2020), and molecular imaging (Li et al., 2018). Specifically, the platelet membrane has inspired the design of many functional nanomaterials owing to its unique surface moieties (Kunde and Wairkar, 2021), such as CD47, which can reduce phagocyte uptake and glycoprotein VI (GPVI) that can facilitate collagen binding (He et al., 2018; Rao et al., 2017). This suggests that platelet membranes can be harnessed to decorate nanomaterials as a promising strategy to prevent nanomaterial phagocytosis.

Herein, we aimed to use platelet membranes to envelope biomaterials to endow them with biomimetic properties. Bioactive glass (BG) has been recognized as a promising biomaterial for hard and soft tissue regeneration. The ionic dissolution products from BG, such as calcium, silicon, and phosphate ions, can induce, trigger osteoblast differentiation, and promote angiogenesis, all of which are essential for stimulating osteogenesis. Nevertheless, the *in vivo* therapeutic efficacy of BG is severely hindered by its dose-dependent cytotoxicity and phagocytic clearance (Zheng et al., 2021). Therefore, a platelet membrane was used in this studyto coat the active glass to facilitate bone regeneration. By virtue of the immune evasion capability of coated platelet membrane, as-prepared PBG can significantly reduce the uptake by macrophages and regulate the immune environment, as evidenced by the downregulated inflammatory-related gene expression. Moreover, the PBG-modulated immune environment effectively triggered robust osteogenic differentiation of human bone marrow stem cells (hBMSCs) compared to BG without membrane cloaking. Taken together, these findings demonstrate that PBG could serve as a potential immunomodulatory material devised for improved bone regeneration.

## Results and discussion

### Platelet membrane coating on bioactive glass

**Figure 1:**
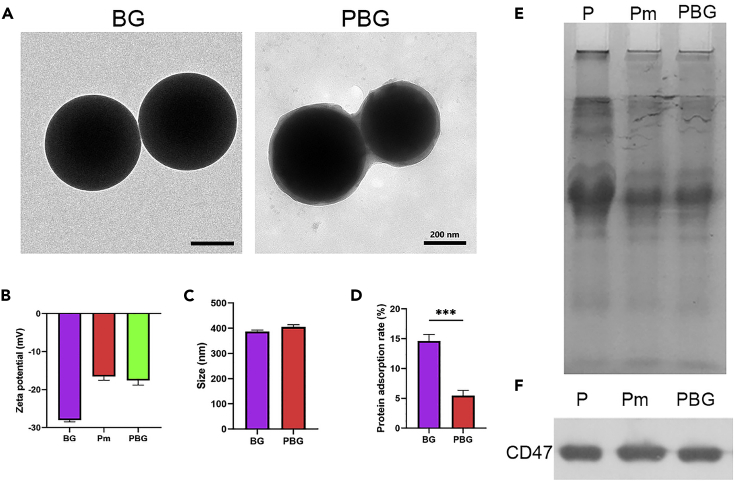
Characterization of PBG (A) Representative TEM images of BG and PBG. Scale bar, 200nm. (B) Surface charge of BG, platelet membrane (Pm), and PBG measured by DLS. (C) Particle diameter. (D) Protein adsorption analysis via BCA assay kit. (E) SDS-PAGE analysis of proteins presents on platelet (P), Pm, and PBG. (F) Western blotting analysis of CD47 present on P, Pm, and PBG. Values represent the mean ± SEM Statistical significance was analyzed by one-way ANOVA and indicated by ∗∗∗ (p < 0.0005).

As shown in Figure 1A, the transmission electron microscopy (TEM) images indicate that BG possessed typical round-like morphology with a particle size of around 400 nm (Figure 1A). After membrane coating, PBG displayed a core-shell structure with a uniform membrane shell at 10-20 nm in-depth (Figure 1A). Meanwhile, dynamic light scattering (DLS) measurement revealed that the average hydrodynamic particle size of PBG particles was ∼19 nm larger than that of BG (Figure 1C), which is in alignment with the TEM results. The successfully developed PBG particles were further confirmed by the change in the surface zeta potential. As shown in Figure 1B, the surface potential of BG particles (−28.1 ± 0.4 mV) increased after the membrane coating (−17.6 ± 0.7 mV). Note that negatively charged PBG possessed an equivalent surface zeta potential to platelet membrane-derived vesicles (Zhuang et al., 2020). Translocation of platelet membrane onto BG was further examined by sodium dodecyl sulfate-polyacrylamide gel electrophoresis (SDS-PAGE) analysis (Figure 1E), which showed no significant difference in the protein profiles between the purified platelet membrane and PBG. Notably, CD47, also known as the “don’t-eat-me” signal, is responsible for inhibiting phagocytosis through its effect on signal regulatory protein alpha (SIRPα). The expression of CD47 was found at a near equivalent degree on PBG compared to that of platelet membrane through the Western blotting analysis (Figure 1F), indicating the preservation of key membrane protein after coating. Moreover, BG showed remarkably higher surface protein adsorption than PBG (Figure 1D), suggesting that the phospholipid bilayer of the platelet membrane can effectively protect BG from protein adsorption. These results collectively indicate the successful coating of the natural platelet membrane onto BG particles.

### Evaluation of cytotoxicity and cellular uptake

**Figure 2:**
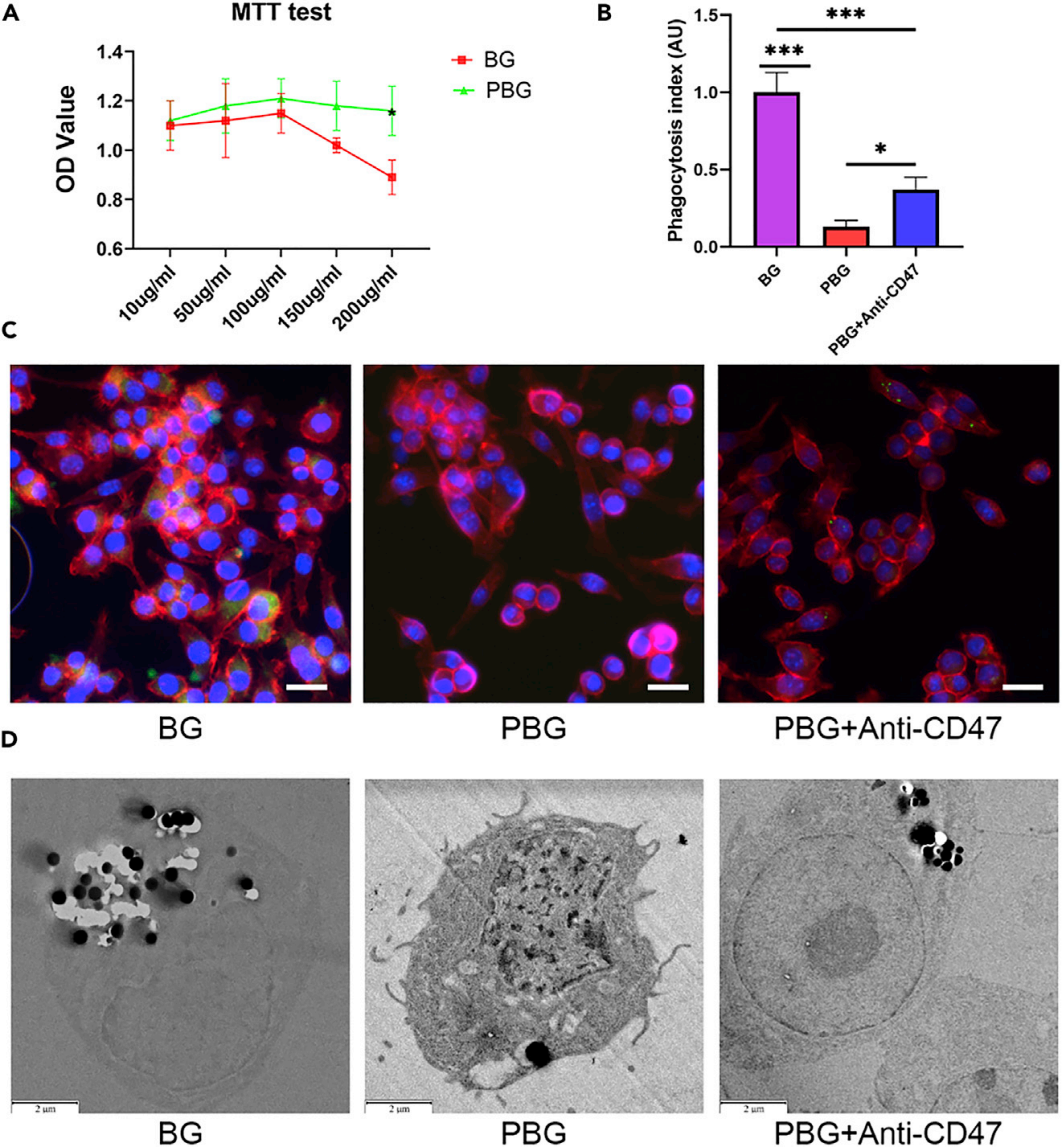
Biocompatibility and phagocytosis of PBG in macrophages (A) MTT test of particle-treated macrophages viability on day 1. (B) Quantification of phagocytized BG, PBG, and anti-CD47 antibody blocked PBG by macrophages. The phagocytosis index is defined as the mean fluorescence intensity per field from six representative images. (C) Confocal images of macrophages cultured with particles. Blue, DAPI-labeled nucleus; Green, FITC-labeled particles; Red, phalloidin-labeled cytoplasm. Scale bar, 20 μm. (D) TEM images of particles internalized by macrophages. Scale bar, 2 μm. Values represent the mean ± SEM Statistical significance was analyzed by one-way ANOVA and indicated by ∗(p < 0. 05), ∗∗∗ (p < 0.0005).

After administration, nanoparticles are rapidly exposed to and recognized by MPS (Gustafson et al., 2015), in which macrophages play a major role in nanoparticle uptake and clearance. Engineering particle surface and biological characteristics can help alter the behaviors of macrophages, including phagocytic recognition, clearance, cellular processing, and toxicological fates. To understand how the platelet membrane shield influences these interactions, we used the murine macrophage cell line RAW 264.7 as a model phagocyte to evaluate its different responses to BG and PBG. As shown in Figure 2A, the dose-dependent cytotoxicity of PBG against macrophages was negligible, as over 90% cell viability was detected after 24 h of incubation, demonstrating the biocompatibility of as-prepared PBG particles. In comparison, BG particles exhibited no significant cytotoxicity toward macrophages under low concentrations (<100 μg/mL). At the same time, the suppression of cell proliferation was significant in a dose-dependent manner under higher concentrations (>100 μg/mL), consistent with the previous reports (Xie et al., 2019). Considering the dose-dependent toxicity of BG particles, the particle concentration of 100 μg/mL was particularly selected to study the cellular performances of BG and PBG in the following experiments.

More interestingly, coating the platelet membrane on BG led to significantly reduced endocytosis. To evaluate endocytosis, particles were labeled with FITC and then used to treat macrophages. As shown in Figure 2C, the percentage of FITC-positive cells decreased considerably in the PBG group compared with the BG group, demonstrating that the platelet membrane inhibits the uptake of BG particles by macrophages, which should mitigate the subsequent side effects. Considering the importance of the CD47-derived “don’t eat me” signal in reducing cellular uptake, we next added an anti-CD47 antibody to neutralize their activity on the membrane surface, which significantly increased PBG internalization (Figures 2B and 2D). This provides direct evidence of the CD47-dependent shield effect of platelet membrane coating. It is worth noting that macrophages still took up more BG particles than anti-CD47 neutralized PBG (Figures 2C and 2D), indicating that platelet membrane can reduce the uptake through various pathways (Hu et al., 2015).

Consistent with the confocal results, the TEM images showed less particle internalization in the PBG group in comparison with the BG group (Figures 2D and S1). Intriguingly, a large number of BG particles were found to locate in the cytosol of macrophages, whereas PBG was wrapped by lysosomes. Following phagocytosis, vesicles containing the foreign material may fuse with lysosomal compartments and then undergo enzyme-catalyzed hydrolysis. Under low particle concentrations, macrophages can process the ingested BG particles within the phagosome vesicles (Gómez-Cerezo et al., 2018). However, the excessive uptake of BG particles leads to unaffordable accumulation in macrophages, which results in the destruction of phagosomes and a decrease in cell viability (Gustafson et al., 2015). The platelet membrane endows BG particles with a stealthy surface to escape from the MPS, not only through CD47-SIRPα interaction but also via protein adsorption reduction (Figures 2B and 2C) (Hadjidemetriou et al., 2019; Zou et al., 2020). The engagement of SIRPα by CD47 brings a downregulatory signal that inhibits immunocyte phagocytosis (Barclay and van den Berg, 2014). In addition, once in contact with biological fluids, biomolecules can be quickly adsorbed onto the surface of particles to form a “protein corona,” which would strongly affect cellular interactions and promote internalization (Francia et al., 2019). Thus, the reduced protein adsorption of PBG (Figure 2A) may mitigate the formation of “protein corona” and thereby decrease cellular internalization. As a result, the suppression of cell viability in the BG group should result from the massive uptake of BG particles by macrophages. At the same time, platelet membrane coating can effectively limit cellular uptake.

### Effects of particle uptake on macrophage polarization

**Figure 3:**
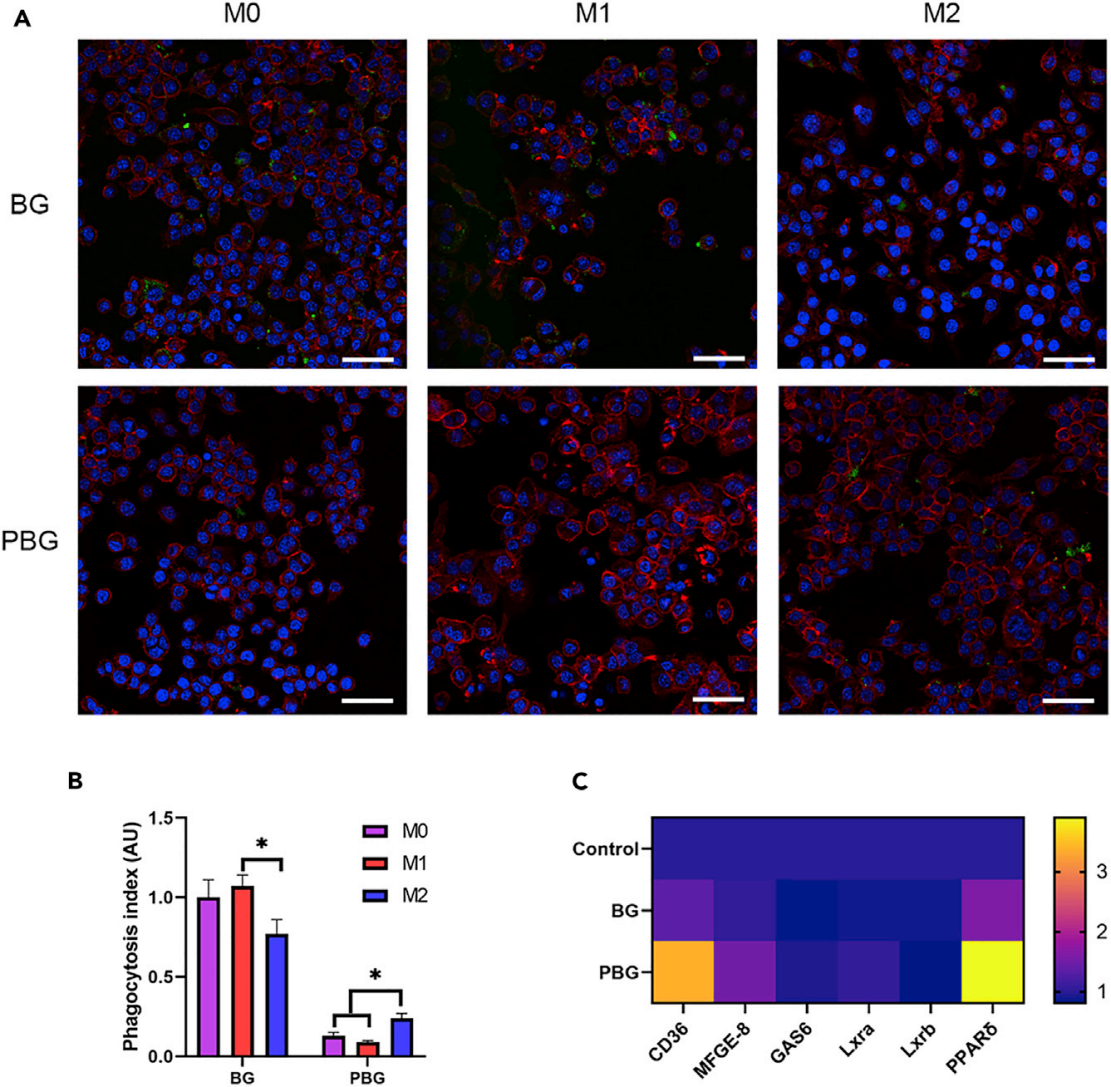
Phagocytosis of PBG in different polarized macrophages and efferocytosis-related gene expressions in macrophages (A) Confocal images of particles internalized by different phenotypes of macrophages after 30 min. Blue, DAPI-labeled nucleus; Green, FITC-labeled particles; Red, phalloidin-labeled cytoplasm. Scale bar, 50 μm. (B) Quantification of internalized particles. The phagocytosis index is defined as the mean fluorescence intensity per field from six representative images. (C) Expression of efferocytosis-related genes after macrophages cultured with BG and PBG for 2 days. Values represent the mean ± SEM Statistical significance was analyzed by one-way ANOVA and indicated by ∗(p < 0. 05).

It is well recognized that macrophages possess significant plasticity, which polarizing toward a spectrum of phenotypes in a reversible manner under different stimuli to achieve unique immune functions, and the two ends of this spectrum are referred to as the M1-like and M2-like phenotypes (Franz et al., 2011; Xiao et al., 2020). M1 macrophages have been reported to be microbicidal and pro-inflammatory through enhanced phagocytosis of bacterial pathogens with up-regulated surface complement receptors and increased complement secretion (Lee et al., 2016). In contrast, M2 macrophages facilitate the clearance of apoptotic cells to resolve inflammation and maintain tissue homeostasis (Boada-Romero et al., 2020). Thus, to study whether the functional differences between M1 and M2 macrophages and unpolarized M0 affect particle clearance, we tested the clearance of particles in differentially polarized macrophages. As shown in Figure 3A, M2 macrophages showed reduced BG internalization and increased PBG uptake capacity compared to the M1 and M0 counterparts, as evidenced by the intracellular green fluorescence signals, which are similar to the previous reports on CD47-modified particles (Herd et al., 2015; MacParland et al., 2017). Although CD47-SIRPα interaction predominantly regulates phagocytosis, the distribution pattern of CD47 may be altered during incubation and reduce the binding avidity of CD47 to SIRPα, which facilitates the apoptotic cell clearance by macrophages (Lv et al., 2015). The clearance of apoptotic cells, a process termed “efferocytosis,” has been reported to induce M2 macrophage activation and interleukin-10 (IL-10) production (Röszer, 2017); the activated M2 macrophages are able to reduce inflammation, aid tissue regeneration, and internalize apoptotic cells (Lawrence and Natoli, 2011; Roszer, 2015). Consistent with this observation, we noted that macrophages treated with PBG showed significantly higher expression of efferocytosis-related genes (CD36, MFGE-8, and PPARδ) and increased the secretion of IL-10 compared to the control group and BG group, demonstrating that PBG particles can be uptaken by M2 macrophages through efferocytosis.

### Immunomodulatory capacity of platelet membrane-coated bioactive glass

**Figure 4:**
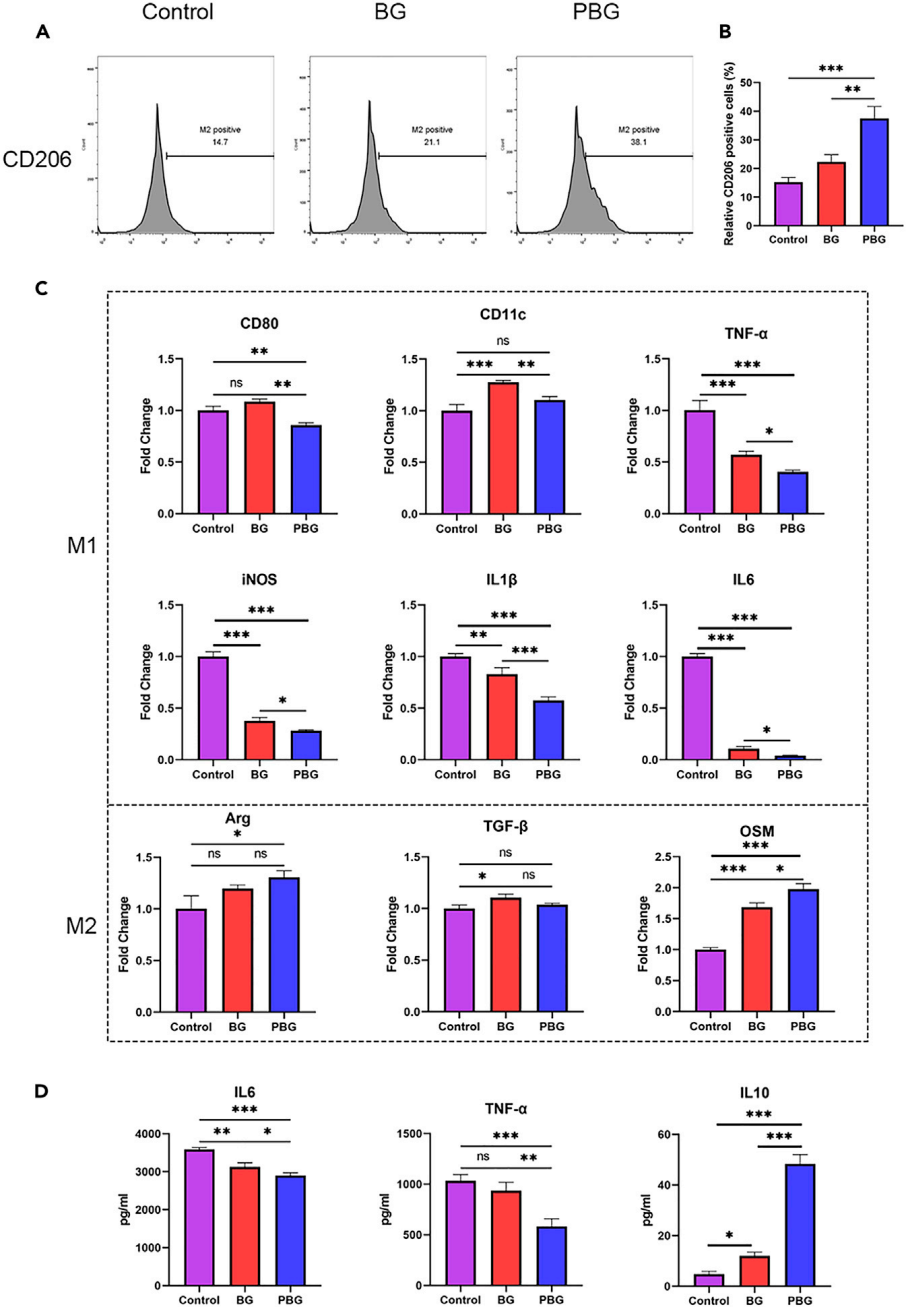
Immunomodulation effect of PBG (A) Flow cytometry results of macrophages cultured with BG and PBG after 2 days. Macrophages were stained with M1 marker F4/80 and M2 marker. (B) Positive cell percentage of M1 and M2 phenotypes. (C) Expression of inflammation-related genes in macrophages cultured with BG and PBG after 2 days. (D) ELISA assay of inflammatory cytokines release on day 2. Values represent the mean ± SEM Statistical significance was analyzed by one-way ANOVA and indicated by ns (p ≥ 0. 05), ∗(p < 0. 05), ∗∗(p < 0. 005), ∗∗∗ (p < 0.0005).

Tissue regeneration requires proper modulation of inflammatory cells, as an unfavorable immune environment leads to prolonged and impaired the resolution of inflammation (Chen et al., 2018; Liu et al., 2020). To evaluate the immunomodulatory capacity of PBG particles, LPS-stimulated M1 macrophages were used as inflammatory cells to mimic the *in vivo* acute inflammatory state (Chen et al., 2016; Gao et al., 2018). The flow cytometry analysis showed that PBG induced elevated CD206 expression in M1 macrophages compared with BG and control groups (Figures 4A and 4B), demonstrating that macrophages were polarized to the M2 phenotype after being stimulated by PBG. Consistently, the expression of pro-inflammatory genes, including CD80, IL-1β, TNFα, iNOS, and IL-6, were significantly downregulated. In contrast, the anti-inflammatory genes Arg and OSM were upregulated in M1 macrophages treated with PBG (Figure 4C). These results indicated that PBG facilitated the M1-to-M2 phenotype switch of macrophages as compared to that treated with BG particles. Previous studies have demonstrated that BG particles could induce macrophages to polarize toward the M2 phenotype through direct cellular interactions (Li et al., 2021) or ion release (Zhao et al., 2018). Benefitting from the coated platelet membrane, PBG exhibited prolonged circulation time to release ionic products without damaging phagocyte organelles and, more importantly, promote the production of pro-resolution cytokines. As shown in Figure 4D, compared with the BG group, PBG significantly decreased the production of cytokines TNFα by 22% and IL6 by 43%. In contrast, the secretion of IL10, one of the most important anti-inflammatory cytokines identified as a modulator of the inflammatory reaction, was remarkably increased (Figure 4D), which is consistent with the previous reports (Hovsepian et al., 2013).

**Figure 5:**
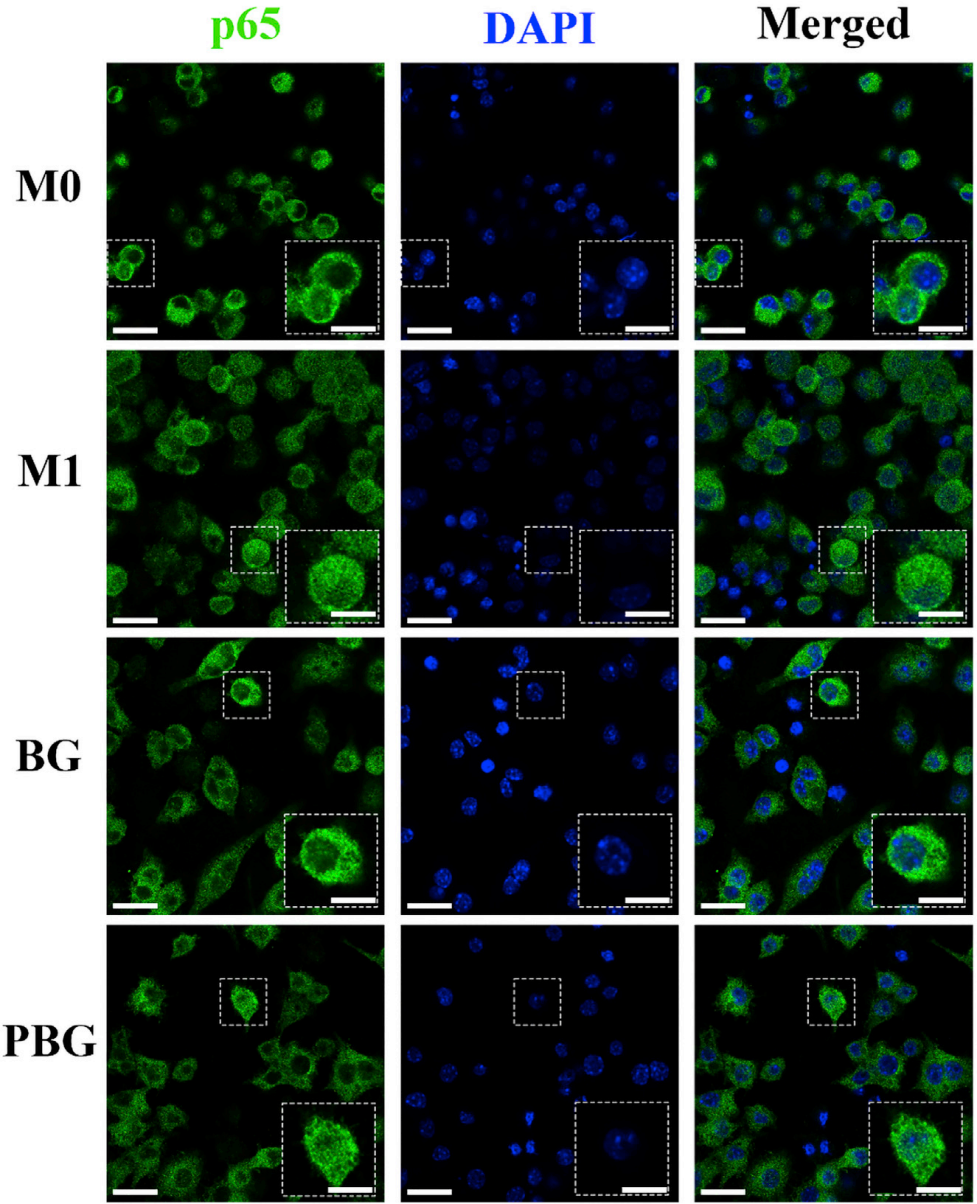
Immunomodulation effect of PBG Immunofluorescent staining of NF-ƙB p65 translocation in macrophages cultured with standard medium (M0), normal medium plus 100 ng/ml LPS (M1), M1 supplemented with 100 μg/mL BG (BG), and M1 supplemented with 100 μg/mL PBG (PBG) for 2 days. Scale bar, p65, DAPI, Merged, 30 μm; High-Mag, 15 μm.

Considering the essential role of the NF-κB signal pathway related to M1 polarization of macrophages, we further evaluated the changes in the activation status of transcription factor NF-κB p65, a most abundant form of NF-κB activated by the canonical pathway (DeFelice et al., 2019). As shown in Figure 5, strong green fluorescence was observed in the nucleus of M1 macrophages without any treatment, implying the translocation of NF-κB from the cytoplasm into the nucleus after activation. Interestingly, nuclear NF-κB localization significantly decreased after PBG treatment (Figure S2), which is consistent with the M1-to-M2 transition in macrophages treated with PBG (Figure 4A).

### The osteoimmunomodulatory effects of platelet membrane-coated bioactive glass

**Figure 6:**
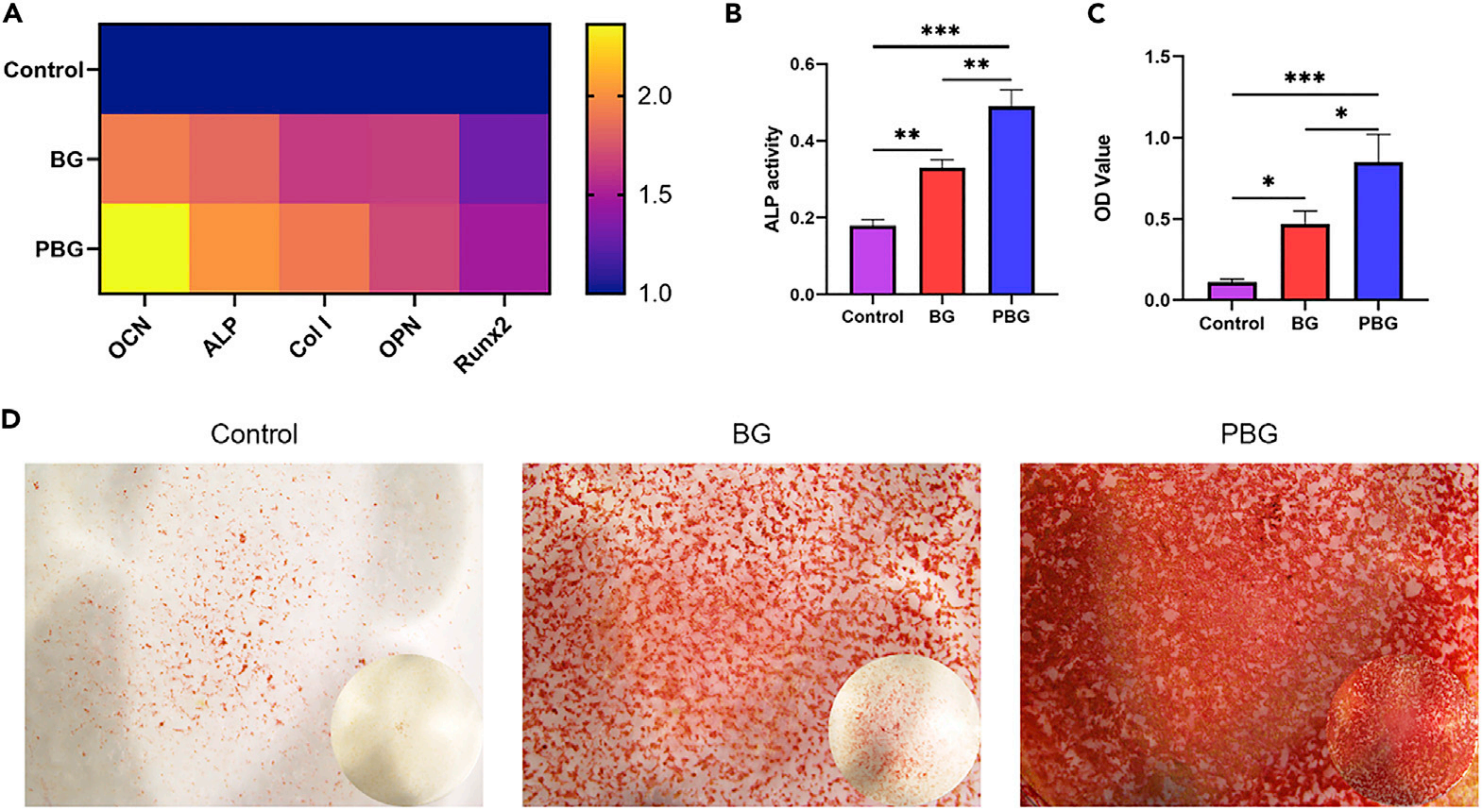
Osteogenic effect of PBG-conditioned macrophages (A–D) mRNA expression of osteogenesis-related genes in hBMSCs cultured with BG-CM and PBG-CM after osteogenic induction for 14 days (B) ALP activity of hBMSCs grown for 7 days in BG-CM and PBG-CM with osteogenic supplements. (C) Quantitative analysis results of Alizarin Red S staining and (D) Alizarin Red S staining of hBMSCs cultured with BG-CM and PBG-CM for 14 days. The inset of (D) shows the whole-cell culture well. Values represent the mean ± SEM Statistical significance was analyzed by one-way ANOVA and indicated by ∗(p < 0. 05), ∗∗(p < 0. 005), ∗∗∗ (p < 0.0005).

Studies on osteoimmunology have revealed a close relationship between immune response and bone regeneration by sharing a few cytokines, transcription factors, receptors, and signaling molecules (Okamoto et al., 2017). An ideal osteoimmunomodulatory biomaterial for bone regeneration is expected to modify the local immune environment into one suitable for osteogenesis (Chen et al., 2016). Especially, a biomaterial that induces an M1-to-M2 switch in the macrophage population is considered to benefit bone regeneration (Xiao et al., 2022).To understand whether the PBG could create a more favorable immune environment to support the subsequent bone regeneration, the osteogenic differentiation of human bone marrow stromal cells (hBMSCs) in response to conditioned medium (CM) produced by material-stimulated macrophages was assessed. The mRNA levels of osteogenesis-associated genes OPN, OCN, ALP, and Runx2 in hBMSCs treated with CM for 14 days were shown in Figure 6A. Compared to the BG-CM treated group, PBG-CM significantly enhanced the osteogenic differentiation of hBMSC, as evidenced by the upregulated expression of OPN, OCN, and Runx2. Accordingly, ALP activity was remarkably upregulated in hBMSCs treated with PBG-CM (Figure 6B), indicating that PBG can facilitate early osteogenesis. Moreover, the deposition of mineralized nodules by bone-forming cells was examined using Alizarin Red S staining. As shown in Figure 6D, more pronounced mineralized nodules were observed in the PBG-CM treated group, as compared with the BG-CM group and the control group, in accordance with the quantification results in Figure 6C. In consistent with the PCR results, the immunofluorescent staining images showed that PBG-CM treated hBMSCs has stronger green fluorescent signals and intensities in ALP (7.60) and Col-I (9.21) than control and BG group (Figure S3). These results demonstrated that the PBG-modified immune environment could enhance the osteogenesis of hBMSCs.

Previous studies have revealed the osteogenic effect of CM from BG extracts rather than from BG particles (Xie et al., 2019; Zhang et al., 2016; Zhao et al., 2018). This could be owing to the dysregulated immune environment resulting from the excessive BG particle uptake by the local phagocytes, which consequently impairs osteogenesis. In contrast, the ionic environment created by BG extracts showed no distinct impact on macrophage function and structural integrity (Hench et al., 2012). As previously noted, as-prepared PBG particles showed excellent immune evasion properties. They avoided the induction of dysregulated immune response, which retains the bioactive function by continuously releasing bioactive ions to promote osteogenesis. In addition, PBG particles can activate more M2 macrophages to induce stronger more robustnic differentiation and upregulate the secretion of IL-10, which may be active in the p38/MAPK signaling pathway to promote osteogenic differentiation (Kraynak et al., 2020; Pajarinen et al., 2019).

### Conclusion

In this study, platelet membrane-coated bioactive glass has been successfully prepared. The platelet membrane coating endowed BG with immune evasion properties and elicited a beneficial effect of regulating the inflammatory response of macrophages. The immune environment induced by PBG enhanced osteogenesis compared to that of BG. The excellent immune evasion ability, immunomodulatory capacity, and superior osteogenesis properties suggested that platelet membrane coating could be used as a potential technique to alleviate foreign body reactions and enhance the clinical performance of biomaterials for bone regeneration.

### Limitations of the study

Future studies will be conducted for the *in vivo* validation of the immunomodulation and osteogenesis effect of PBG. However, owing to the COVID restriction, we cannot perform the *in vivo* study in our current project.

## STAR★Methods

### Key resources table

**Table:** 

REAGENT or RESOURCE	SOURCE	IDENTIFIER
**Antibodies**		

Anti-CD47 antibody	Abcam	ab300435
anti-rabbit IgG IRDye 800	Rockland	RRID:AB_828189
Alkaline Phosphatase, Tissue Non-Specific antibody	Abcam	RRID:AB_10862036
Rabbit Anti-Collagen I Polyclonal Antibody	Abcam	RRID:AB_731684
Donkey Anti-Rabbit IgG H&L (Alexa Fluor® 488)	Abcam	RRID:AB_2636877
Alexa Fluor™ 594 Phalloidin	Invitrogen	A12381

**Biological samples**		

Human blood	Australian Red Cross Blood Bank	N/A

**Chemicals, peptides, and recombinant proteins**		

EDTA	Sigma Aldrich	60-00-4
prostaglandin E1	Sigma Aldrich	745-65-3
Tetraethyl orthosilicate	Sigma Aldrich	78-10-4
triethylphosphate	Sigma Aldrich	78-40-0
Calcium nitrate tetrahydrate	Sigma Aldrich	13477-34-4
Dodecylamine	Sigma Aldrich	124-22-1
Pierce™ Protease Inhibitor Tablets, EDTA-free	Thermo Scientific	A32965
Fluorescein isothiocyanate	Sigma Aldrich	F7250
Odyssey Blocking Buffer	LI-COR Biosciences	927–40100
Dulbecco’s modified Eagle’s medium	Gibco	11885092
Fetal bovine serum	*In Vitro* Technologies	

**Critical commercial assays**		

Pierce BCA Protein Assay Kit	Life Technologies	23225

**Experimental models: Cell lines**		

RAW264.7		
hBMSCs		

**Oligonucleotides**		

Primers for CD11c forward: ACTTCACGGCCTCTCTTCC	This paper	N/A
Primers for CD11c reverse: CACCAGGGTCTTCAAGTCTG	This paper	N/A
Primers for TNF-α forward: CTGAACTTCGGGGTGATCGG	This paper	N/A
Primers for TNF-α reverse: GGCTTGTCACTCGAATTTTGAGA	This paper	N/A
Primers for IL-1β forward: GGATGATGATGATAACCTGC	This paper	N/A
Primers for IL-1β reverse: CATGGAGAATATCACTTGTTGG	This paper	N/A
Primers for IL-6 forward: ATAGTCCTTCCTACCCCAATTTCC	This paper	N/A
Primers for IL-6 reverse: GATGAATTGGATGGTCTTGGTCC	This paper	N/A
Primers for iNOS forward: CAGAAGTGCAAAGTCTCAGACAT	This paper	N/A
Primers for iNOS reverse: GTCATCTTGTATTGTTGGGCT	This paper	N/A
Primers for IL-10 forward: CTGGGTGAGAAGCTGAAGAC	This paper	N/A
Primers for IL-10 reverse: GACACCTTGGTCTTGGAGCTTA	This paper	N/A
Primers for CD80 forward: AAAAGAAGGAAAGAGGAACGTATGAA	This paper	N/A
Primers for CD80 reverse: CCGGAAGCAAAGCAGGTAATC	This paper	N/A
Primers for OSM forward: ACGGTCCACTACAACACCAG	This paper	N/A
Primers for OSM reverse: CCATCGTCCCATTCCCTGAAG	This paper	N/A
Primers for TGF-β forward: CAGTACAGCAAGGTCCTTGC	This paper	N/A
Primers for TGF-β reverse: ACGTAGTAGACGATGGGCAG	This paper	N/A
Primers for CD36 forward: TCGGAACTGTGGGCTCATTG	This paper	N/A
Primers for CD36 reverse: CCTCGGGGTCCTGAGTTATATTTTC	This paper	N/A
Primers for MFGE-8 forward: GGACATCTTCACCGAATACATCTGC	This paper	N/A
Primers for MFGE-8 reverse: TGATACCCGCATCTTCCGCAG	This paper	N/A
Primers for GAS6 forward: TCTTCTCACACTGTGCTGTTGCG	This paper	N/A
Primers for GAS6 reverse: GGTCAGGCAAGTTCTGAACACAT	This paper	N/A
Primers for Lxrα forward: TCGCCATCAACATCTTCTCAG	This paper	N/A
Primers for Lxrα reverse: GTGTGGTAGGCTGAGGTGTAA	This paper	N/A
Primers for Lxrb forward: TCCATCAACCACCCCCACGAC	This paper	N/A
Primers for Lxrb reverse: CAGCCAGAAAACACCCAACCT	This paper	N/A
Primers for PPARδ forward: CTCCTGCTGACTGACAGATG	This paper	N/A
Primers for PPARδ reverse: TCTCCTCCTGTGGCTGTTC	This paper	N/A
Primers for OCN forward: TCACACTCCTCGCCCTATTG	This paper	N/A
Primers for OCN reverse: GAAGAGGAAAGAAGGGTGCC	This paper	N/A
Primers for Col-I forward: CCCTGGAAAGAATGGAGATGAT	This paper	N/A
Primers for Col-I reverse: ACCATCCAAACCACTGAAACCT	This paper	N/A
Primers for OPN forward: TCACCAGTCTGATGAGTCTCACCATTC	This paper	N/A
Primers for OPN reverse: TAGCATCAGGGTACTGGATGTCAGGTC	This paper	N/A
Primers for RUNX2 forward: CATGGCGGGTAACGATGAA	This paper	N/A
Primers for RUNX2 reverse: AGACGGTTATGGTCAAGGTGAAA	This paper	N/A
Primers for ALP forward: TCTTCACATTTGGTGGATAC	This paper	N/A
Primers for ALP reverse: ATGGAGACATTCTCTCGTTC	This paper	N/A

**Software and algorithms**		

Graphpad Prism	Graphpad	https://www.graphpad.com/
FlowJo	FlowJo	https://www.flowjo.com/
ImageJ	National Institutes of Health	https://imagej.nih.gov/ij/

### Resource availability

#### Lead contact

Further information and requests for resources should be directed to and will be fulfilled by the lead contact, Yin Xiao (yin.xiao@griffith.edu.au).

#### Materials availability

This study did not generate new unique reagents.

### Experimental model and subject details cell culture

The murine-derived macrophage cell line RAW 264.7 was maintained in Dulbecco’s modified Eagle’s medium (DMEM; Gibco, Life Technologies Pty Ltd., Australia) supplemented with 10% (v/v) heat-inactivated fetal bovine serum (FBS, *In Vitro* Technologies, Australia), and 1% (v/v) penicillin/streptomycin (P/S, Gibco, Life Technologies Pty Ltd., Australia) in a humidified incubator containing 5% CO_2_ at 37°C. Human bone marrow stromal cells (hBMSCs) were isolated with bone marrow samples obtained from patients undergoing elective knee replacement surgery with informed consent at the Department of Orthopedics, Prince Charles Hospital. All procedures were approved by the Ethics Committee of Queensland University of Technology (ethics approval number: NO. 1400001024). The bone marrow samples were flushed using DMEM. Then, the samples were gently transferred into a T75 culture flask. After culture for one1 day, the culture medium was replaced. The hBMSCs were cultured with 10% FBS and 1% (v/v) P/S at 37°C in a humidified incubator containing 5% CO_2_. Passages 5–7 of hBMSCs were used in this study.

### Method details

#### Preparation of platelet membrane

Human blood anti-coagulated with 1.5 mg/mL EDTA was kindly provided by the Australian Red Cross Blood Bank (Human ethics approval number: 2,021,000,021) and processed for platelet collection approximately 2 h after blood collection. The blood samples were centrifuged at 100g for 20 min at room temperature to separate red blood cells and white blood cells to isolate platelets. The resulting platelet-rich plasma (PRP) was then centrifuged at 100g for 20 min to remove the remaining blood cells. PBS with 1 mM of EDTA and 2 uM of prostaglandin E1 (PGE1) was added to the purified PRP to prevent platelet activation. Platelets were then pelleted by centrifugation at room temperature at 800g for 20 min. The supernatant was discarded, and the platelets were resuspended in PBS containing 1 mM of EDTA and mixed with Pierce Protease Inhibitor Tablets. 1 mL aliquots of platelet solution containing ∼2×10^9^ platelet were prepared and used to cloak 2 mg of BG particles.

The Platelet membrane was derived by a repeated freeze-thaw process. Aliquots of platelet suspensions were first frozen in liquid nitrogen, thawed at room temperature, and pelleted by centrifugation at 4000*g* for 3min. After three repeated washes with PBS solution mixed with protease inhibitor tablets, the pelleted platelet membranes were suspended in water and sonicated in a capped glass vial for 5 min using a FS30D bath sonicator at a frequency of 42kHz and a power of 100W.

#### Preparation and characterization of platelet membrane-coated bioactive glass (PBG)

A bioactive glass (BG) particle with a size of around 400 nm was synthesized by a sol-gel method (Gao et al., 2017). Firstly, dodecylamine was dissolved in a mixture of 80 mL of ethanol and 25 of deionized water. After the complete dissolution, 16 mL of tetraethyl orthosilicate was added to the above solution and stirred for 1 h. Then, triethylphosphate and calcium nitrate tetrahydrate were added in the proportions at 30 min intervals while stirring at 40°C. The resulting mixture was stirred vigorously for 3 h until a white precipitate was formed. The synthesized products were centrifuged, sequentially washed three times with distilled water and ethanol, and then freeze dried for 24 h. The final products were obtained by sintering in air at 650°C for 3 h to remove residual DDA and organic components. The composition of BG was 60% SiO_2_, 36% CaO, and 4% P_2_O_5_ (mol/mol). For fluorescently labeled BG, Fluorescein isothiocyanate (FITC) was loaded into the BG at 0.1 wt %. The mixture was then stirred in the open air for 3 h and washed thrice in Milli-Q water. Platelet membrane-coating was then accomplished by sonication using an FS30D bath sonicator at a frequency of 42kHZ and a power of 100W for 3 min. The size and the surface zeta potential of replicate PBG samples (n = 3) were quantified using dynamic light scattering (DLS, Malvern). The structure of PBG was captured using transmission electron microscopy (TEM, JEOL 1400) operated at 80 kV.

#### Detection of platelet membrane proteins

PBG was purified from unbound proteins or membrane fragments by centrifuging at 16,000 g in 10% sucrose. Platelets, platelet membrane vesicles, and PBG were then normalized to equivalent overall protein concentration using a Perce BCA Protein Assay Kit. All samples were prepared in lithium dodecyl sulfate (LDS) sample loading buffer (Invitrogen) and run at equivalent protein concentrations on an SDS-PAGE gel (10%–15%) and then separated and transferred to a nitrocellulose membrane (Merck Millipore, Billerica, MA). The membrane was blocked using Odyssey Blocking Buffer for 1 h at room temperature and then incubated with primary antibody CD47 along with the appropriate secondaries, anti-rabbit IgG IRDye 800 conjugated secondary antibody (1:10,000) for 1 h at room temperature. The membranes were then scanned/analyzed using an Odyssey Infrared Imaging System and Image Studio software (LI-COR Biosciences) according to the manufacturer’s instructions.

#### Immunomodulation effect of PBG on macrophages

##### Cell viability

*In vitro* cytotoxicity of BG and PBG was evaluated by the MTT assay. RAW264.7 cells were seeded into a 96-well plate (NUNC) at a density of 1×10^5^ cells per 200 μL per well. After the cell was attached to the plate, the medium was refreshed, and cells were treated with BG and PBG for 24 h. The medium was then removed, and the cells were washed with PBS. Fresh culture medium containing MTT solution (0.5 mg/mL Sigma-Aldrich Pty Ltd.) was added to each well and incubated at 37°C for 4 h. The medium was then replaced with 100 μL dimethyl sulfoxide. The absorbance of each well was read at 570 nm by a CLARIOstar Plus plate-reader (BMG labtech).

##### Particle cellular uptake

RAW264.7 cells were seeded in six-well plates at a density of 4×10^5^ cells per well. M1 polarization was induced by 100 ng/mL LPS plus 100 ng/mL IFN-γ; M2 polarization was induced by IL-4 (100 ng/mL). After overnight incubation at 37°C, the medium was refreshed with a culture medium. For the cellular uptake study, different phenotypes of macrophage cells were incubated in replicate wells (n = 3) with FITC-labeled particles (PBG, anti-CD47 blocked PBG, and BG) at 100 μg/mL in a culture medium. After 30 min of incubation at 37°C, the RAW264.7 cells were scraped off the wells and washed three times in PBS to remove non-internalized particles. For fluorescent staining, cells were then fixed and stained with Phalloidin-iFluor 594 (Abcam) and DAPI (Sigma), then observed by confocal laser scanning microscopy (Leica TCP SP5, Leica). For TEM observation, cells were fixed in 2.5% glutaraldehyde, post-fixed with 1% Osmium tetroxide, and dehydrated in gradient concentrations of ethanol (50%, 70%, 90%, 100%) using Pelco Biowave Pro Microwave Tissue Processor (Ted Pella, INC.). Samples were then embedded in gradient resin-ethanol (25%, 50%, 75% and 100%) solution (EPON812, Sigma, Australia), and polymerized in 100% resin at 60°C before ultrafine sectioning using a microtome (EM UC7 Ultramicrotome, Leica). Prior to TEM visualization, ultrathin sections (100 nm) were loaded onto carbon-coated copper grids (standard, ProSciTech Pty Ltd.), and followed by post-staining with 2% uranyl acetate and lead citrate. Samples were observed at an accelerating voltage of 120kV.

#### Response of RAW 264.7 to PBG

To study the immunomodulation effect of PBG on macrophages under inflammatory stimulation, RAW264.7 cells (with or without 24 h stimulation of LPS) were either treated with 100 μg/mL BG or 100 μg/mL PBG for 2 days. Cells cultured on the tissue culture polystyrene (TCP) are used as the control group. Then, cells were washed thoroughly with PBS three times and then cultured with 2mL serum-free DMEM for another 12 h. The conditioned medium (CM) was collected and subjected to centrifugation at the speed of 1000 g for 5 min. The supernatant was then aliquoted and stored at −80°C for further experiments.

#### Quantitative real-time PCR (qRT-PCR)

Total RNA was extracted with TRIzol reagent (Ambion Life Technologies Pty Ltd., Australia) according to the manufacturer’s instructions. RNA concentrations were measured using a NanoDrop spectrophotometer (Thermo Fisher Scientific). Reverse transcription to cDNA was performed using the SensiFAST cDNA Synthesis Kit (Bioline Reagents, Meridian Bioscience Inc., USA). The quantitative real-time reverse-transcription–polymerase chain reaction (qRT-PCR) was performed using a QuantStudio 7 Flex Real-Time PCR System (Applied Biosystems, Thermo Fisher Scientific) according to a two-step PCR protocol (95°C for 2min, 45 cycles of 5 s at 95°C, 10 s at 60°C, and 15 s at 72°C). Primers for *β-actin, CD11c, TNF-α, IL-1β, IL-6, iNOS, IL-10, CD80, TGF-β, CD206, Arginase1, OSM, CD36, MFGE-8, GAS6, Lxra, Lxrb*, and *PPARδ* were designed and purchased from Sigma-Aldrich, Australia. The primer sequences for qRT-PCR in this study are listed in the key resources table. The *β-actin* gene was used as the reference gene to normalize the differences in the amount of total RNA in each sample. All experiments were performed in triplicate for each condition and repeated three times. Data were analyzed according to the comparative ΔΔCt method.

#### Flow cytometry

Flow cytometry analysis was performed to identify the M1 (F4/80) and M2 (CD206) phenotypes of macrophages with anti-mouse F4/80-FITC and anti-mouse CD206-FITC (Sigma). Fluorescence signals were detected and analyzed by a BD FACSCelesta Cell Analyzer (BD).

#### Translocation of NF-ƙB p65 in RAW 264.7

Translocation of transcription factor NF-ƙB p65 in RAW 264.7 cells was visualised by CLSM. Cells were permeabilized with 0.25% Triton X-100 (Merck) in PBS for 10 min and blocked with 4% BSA (BSA, Sigma-Aldrich) in PBS for 1 h. Cells were then incubated with rabbit monoclonal anti-NF-ƙB p65 (D14 × 10^12^, cell signaling) in 1% BSA (1:1000, Cell Signaling Technology, Australia) overnight at 4°C. Afterward, cells were incubated with Alexa Fluor 488 Conjugate anti-rabbit IgG (1:1000, Cell Signaling Technology, Australia) for 1 h in the dark. The nuclei were stained with 4, 6-diamidino-2-phenylindole (DAPI, Molecular Probes) (1:1000) in PBS for 5 min. Images were treated with the ImageJ package, and the translocation of NF-ƙB p65 into the nuclei was determined semi-quantitatively (number per field from five representative images).

#### Osteogenic activity of hBMSCs treated with secreted factors from PBG-conditioned macrophages

The osteogenic media was prepared by adding 10 mM β-glycerophosphate (β-GP, Sigma-Aldrich, Australia), 50 μg/mL L-ascorbic acid 2-phosphate (AA, Sigma-Aldrich, Australia), 10 nM dexamethasone (DEX, Sigma Aldrich, NSW, Australia), 10% (v/v) FBS, 1% (v/v) P/S into DMEM. hBMSCs were seeded in 24-well plates at a density of 2.5×10^4^/cm^2^. After the cell was attached to the plate, culture media was refreshed with the mixture of conditioned media from BG/PBG treated macrophages and fresh osteogenic media at a ratio of 1:1. Media were refreshed every three days.

#### Alizarin Red S staining and quantitative assay

Alizarin Red S Staining was used to highlight mineralized nodules in hBMSCs culture with different CM. After culture for 14 days, cells were fixed in 4% PFA and stained with 2% Alizarin Red S at pH 4.1 for 20 min, then washed and air-dried. Images were acquired with a light microscope. Quantitative analysis of Alizarin Red S staining was performed by eluting the bound stain with 10% cetylpyridinium chloride in 10 mM Na_2_HPO_4_ (pH 7.0) for 1 h. The absorbance of the resulting solution at 405 nm was determined by the CLARIOstar Plus plate-reader (BMG labtech).

#### Osteogenesis-related gene expression in hBMSCs

The expression of osteogenesis-related genes including *OCN, Col-I, OPN, RUNX2,* and *ALP* was evaluated using qRT-PCR as previously described(section Quantitative real-time PCR (qRT-PCR)).

#### Alkaline Phosphatase (ALP) activity in hBMSCs

The ALP activity of hBMSCs was measured using the ALP assay kit (Colorimetric, ab83369, Abcam, Cambridge, UK) following the manufacturer’s protocol. After culture for 7 days, cells were lysed with 200 μL 1% Triton X-100 (Merck, Australia) and centrifuged to collect the supernatant. The supernatant was then incubated with *p*-nitrophenyl phosphate (pNPP) solution in 96-well plates. The resulting solution at 405 nm was determined by the CLARIOstar Plus plate-reader (BMG labtech).

#### Immunofluorescent staining of ALP and Col-I in hBMSCs

After culture for 7 days, cells were fixed in 4% PFA and permeabilized with 0.25% Triton X-100. Then, the samples were incubated with anti-ALP antibody or anti-Col-I antibody overnight. Then the cells were washed with PBS 3 times and incubated with a secondary antibody (Donkey Anti-Rabbit IgG H&L (Alexa Fluor 488)) for 2 h. Afterward, cells were incubated with Alexa Fluor 594 Phalloidin and DAPI and visualized using a laser scanning confocal microscope. The mean fluorescent intensity was calculated by using ImageJ.

### Quantification and statistical analysis

Three independent experiments were performed and at least three parallel samples per test were taken for statistical analysis. All quantitative data were expressed as the mean ± SD. Statistical significance between experimental groups was evaluated using a one-way ANOVA (one-way ANOVA). A value of *p* ＜ 0.05 was considered statistically significant.

## Data Availability

This paper does not report the original code. All data produced in this study are included in the published article and its supplemental information, or are available from the lead contact upon request. Any additional information required to reanalyze the data reported in this paper is available from the lead contact upon request.
