# Supplementary material for: Targeting macrophage endocytosis via platelet membrane coating for advanced osteoimmunomodulation
Source: iScience. 2022 Sep 23;25(10):105196. doi: 10.1016/j.isci.2022.105196 (PMC9556914; doi:10.1016/j.isci.2022.105196)
Supplement: Document S1. Figures S1–S3 [file mmc1.pdf]

iScience, Volume *25*

Supplemental information

Targeting macrophage endocytosis
via platelet membrane coating
for advanced osteoimmunomodulation

Wendong Gao, Lan Xiao, Yuqing Mu, and Yin Xiao

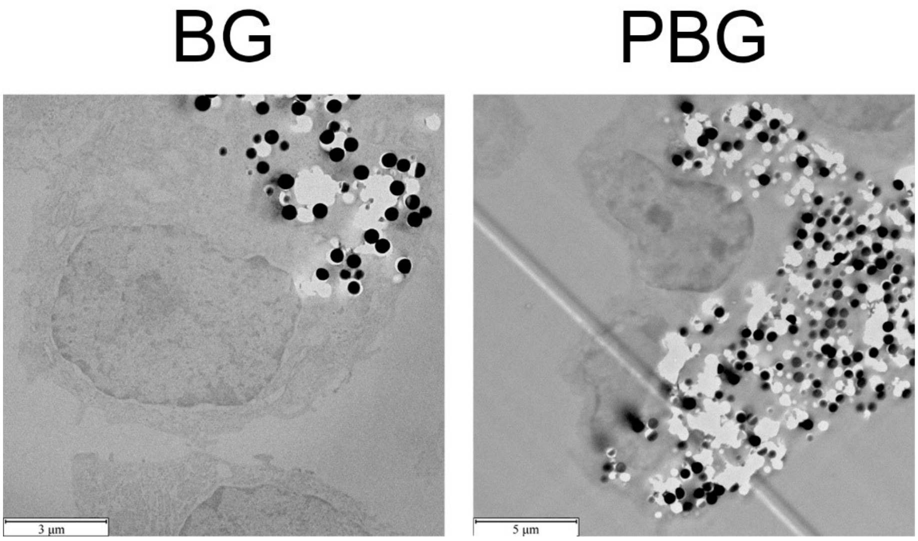

Fig. S1 TEM images of particles internalized by macrophages after culture for 2 days. Related to Fig.2

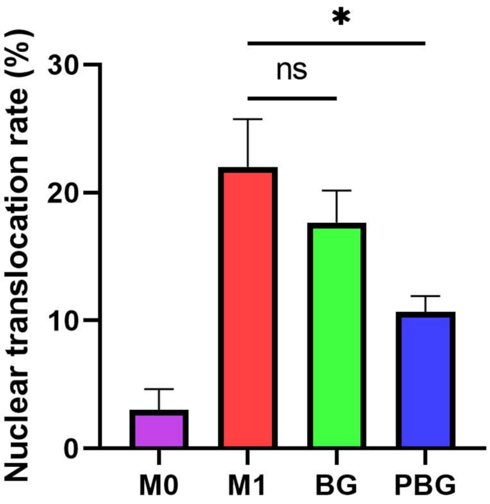

Fig. S2 Nuclear translocation rate is defined by the percentage of macrophages with nuclear NF-kB localization per field from five representative images. Values represent the mean ± SEM. ns: not significant; * Statistically significant, $p < 0.05$. Related to Fig. 5.

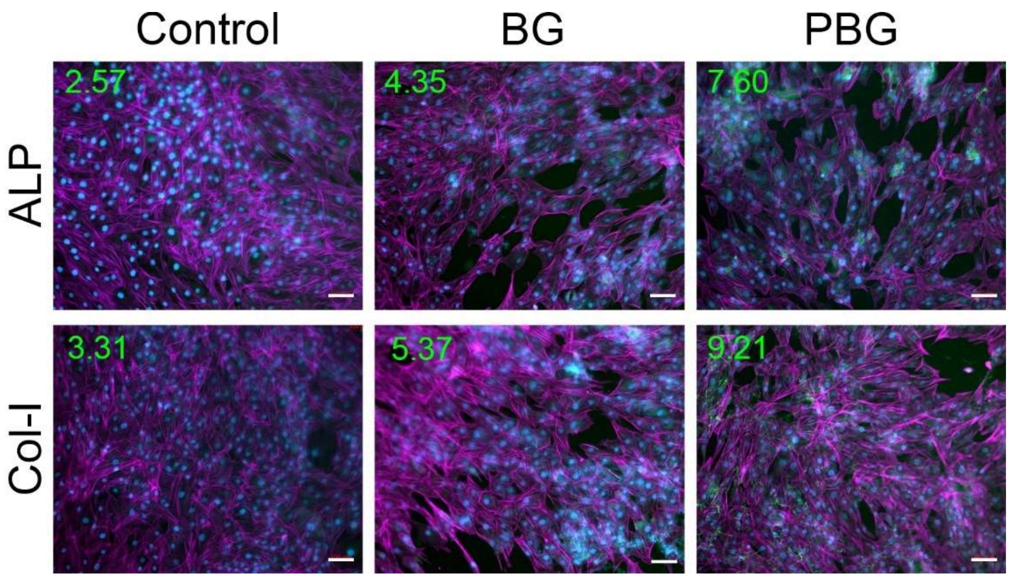

Fig. S3 Representative images of immunofluorescent staining of ALP (green), Col-I (green), Phalloidin (Red) and DAPI (Blue) on hBMSCs on day 7. The numbers on top-left corner indicated the mean fluorescent intensity. Scale bar: 100 μm. Related to Fig. 6.
